# Chronotropic Incompetence Predicts Reduced Survival and Exercise Capacity in Heart Failure: A Meta‐Analysis of Reconstructed Time‐to‐Event Data

**DOI:** 10.1002/clc.70522

**Published:** 2026-11-09

**Authors:** Kiarash Tavakoli, Parham Dastjerdi, Roozbeh Narimani‐Javid, Mohammadreza Babaei, Mehrdad Mahalleh, Amir Hossein Behnoush, Malihe Rezaee, Zainab Humayun, Mohammad Keykhaei, Artur Fedorowski, Masih Tajdini

**Affiliations:** ^1^ Tehran Heart Center, Cardiovascular Diseases Research Institute Tehran University of Medical Sciences Tehran Iran; ^2^ Research Center for Advanced Technologies in Cardiovascular Medicine, Cardiovascular Diseases Research Institute Tehran University of Medical Sciences Tehran Iran; ^3^ Vascular Disease and Thrombosis Research Center Rajaie Cardiovascular Institute Tehran Iran; ^4^ Cardiovascular Intervention Research Center Rajaie Cardiovascular Institute Tehran Iran; ^5^ Division of Cardiology, Department of Medicine Johns Hopkins University School of Medicine Baltimore Maryland USA; ^6^ PGY2 Internal Medicine University of Missouri‐Kansas City School of Medicine Kansas City Missouri USA; ^7^ Department of Cardiology Karolinska University Hospital Stockholm Sweden; ^8^ Department of Medicine Karolinska Institute Stockholm Sweden; ^9^ Cardiac Primary Prevention Research Center, Cardiovascular Diseases Research Institute Tehran University of Medical Sciences Tehran Iran

**Keywords:** chronotropic incompetence, chronotropic response, heart failure, HF, meta‐analysis

## Abstract

**Background:**

The prognostic and functional significance of chronotropic incompetence (CI) across different heart failure (HF) phenotypes remains unclear in previous studies.

**Hypothesis:**

This study compared survival and exercise performance in HF patients with and without CI.

**Methods:**

We systematically searched PubMed, Embase, Scopus, and Cochrane Library until October 2025. Prognosis outcomes were assessed using Cox proportional hazards models based on reconstructed individual patient data. Exercise performance metrics were analyzed as mean differences (MD) using a random‐effects model. Restricted mean survival time analyses quantified differences in life expectancy.

**Results:**

Twenty‐one studies were included. CI was associated with a higher risk of composite cardiovascular events (hazard ratio: 2.75; 95% CI: 2.18–3.48; *p* < 0.001) and all‐cause mortality (hazard ratio: 1.97; 95% CI: 1.64–2.36; *p* < 0.001). Over 2500‐days, patients with CI experienced 410 and 176 fewer event‐free days for composite cardiovascular events and all‐cause mortality, respectively. Patients with CI had significantly lower resting heart rate (HR) (MD: −6.25 bpm; *p* < 0.001), peak HR (MD: −35.12 bpm; *p* < 0.001), and HR reserve (MD: −28.84 bpm; *p* < 0.001). Moreover, the CI subgroup experienced lower exercise duration (MD: −77.68 s; *p*: 0.021), peak workload (MD: −19.15 watts; *p* < 0.001), and peak VO_2_ (MD: −3.34 mL·kg^−1^·min^−1^; *p* < 0.001); with a higher VE/VCO_2_ slope (MD: 2.20; *p* < 0.001). The results remained robust in both reduced and preserved HF phenotypes.

**Conclusion:**

CI is associated with poorer survival and impaired functional capacity. These findings underscore the clinical importance of recognizing and addressing CI in the management of HF patients.

## Practitioner Points

- Heart failure patients with chronotropic incompetence exhibited poorer prognosis outcomes, with approximately 1‐year shorter event‐free survival over a 7‐year follow‐up.
- Heart failure patients with chronotropic incompetence also demonstrated impaired functional capacity reflected in shorter exercise durations, reduced peak exercise output, lower peak VO2, and higher VE/VCO2 slope.
- This survival disadvantage and impaired functional capacity were consistent in both reduced and preserved heart failure phenotypes.

## Graphical abstract

Chronotropic incompetence is associated with worse prognosis and exercise capacity in both heart failure categories. Identification of chronotropic incompetence and finding targeted therapies may improve outcomes.

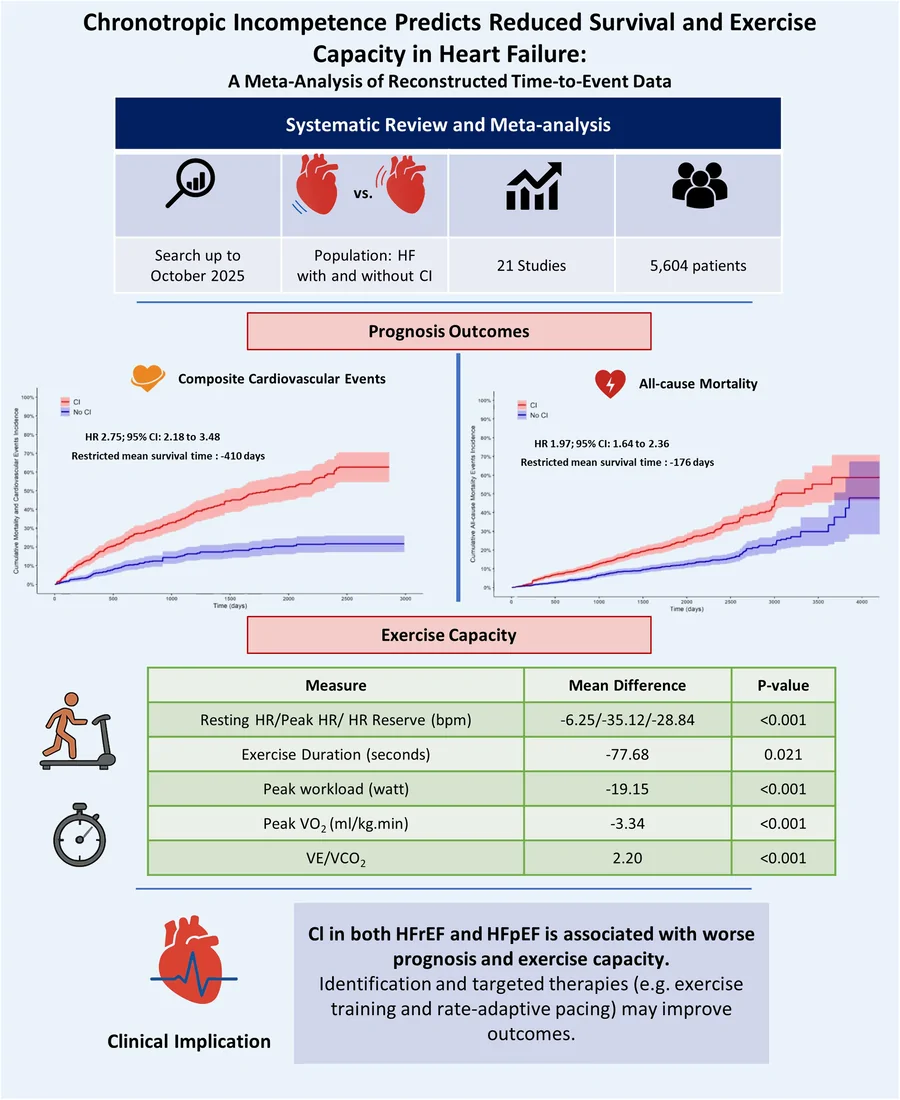

**Abbreviations**
CI: chronotropic incompetence
GRADE: Grading of Recommendations, Assessment, Development, and Evaluations
HF: heart failure
HFpEF: heart failure with preserved ejection fraction
HFrEF: heart failure with reduced ejection fraction mlHRHeart rate
HR: heart rate
IPD: Individual patient data
KM: Kaplan–Meier
MD: mean difference
PROSPERO: International Prospective Register of Systematic Reviews
RMST: restricted mean survival time
ROBINS‐E: Cochrane Risk Of Bias In Non‐randomized Studies‐of Exposure

## 1 Introduction

Chronotropic incompetence (CI), defined as the inability to increase heart rate appropriately in response to increased metabolic demand [1], results from an imbalance between sympathetic and parasympathetic nervous system activity [2]. CI has been associated with reduced exercise capacity, lower quality of life, and poorer prognosis [3, 4]. It is observed across different cardiovascular complications and has even been reported in individuals without any heart disease [2, 5, 6]. CI is particularly common in patients with heart failure (HF), affecting up to 50% of cases [7, 8], which is due to chronic sympathetic overactivation in HF, leading to downregulation and desensitization of cardiac β‐adrenergic receptors [1].

The importance of CI on the prognosis and functional performance of patients with HF has become a topic of interest in recent years. However, there is a lack of consistency between different reports [9, 10, 11, 12]. Moreover, previous meta‐analyses have failed to demonstrate the effect of time in survival analyses of HF patients with CI and lacked sufficient data to fully explore the impact of CI on exercise performance across different HF phenotypes [8]. This systematic review and meta‐analysis aim to comprehensively evaluate the prognostic significance and functional consequences of CI in heart failure patients with reduced ejection fraction (HFrEF) and preserved ejection fraction (HFpEF).

## 2 Materials & Methods

This systematic review and meta‐analysis followed the PRISMA guidelines outlined in Supporting Information S2: Table 1. The study protocol was registered with the International Prospective Register of Systematic Reviews (PROSPERO) under registration code CRD420251039152. Since the analyses relied exclusively on summary data from previously published studies, additional ethical approval was not required.

### 2.1 Search Strategy and Screening

An extensive literature search was conducted across four databases: PubMed, Embase, Scopus, and Cochrane Library, up to October 27, 2025. The search employed the keywords (“Heart failure”) AND (“Chronotropic incompetence”), along with their synonyms, with no restrictions on language or study type. Detailed search strategies for each database are shown in Supporting Information S2: Table 2. Two authors (P.D. and R.N‐J.) independently carried out two rounds of screening, the first based on titles and abstracts and the second on a full‐text review. Discussions with a third author (M.T.) addressed any disagreements regarding study inclusion. Moreover, two authors (M.R. and M.B.) manually reviewed the bibliographies of the included studies and relevant review articles to identify eligible studies for inclusion in the study.

### 2.2 Eligibility Criteria

The inclusion criteria encompassed original articles, including retrospective and prospective cohort studies and randomized controlled trials, that reported Kaplan–Meier (KM) survival curves or at least one exercise performance outcome in patients with HF based on the presence or absence of CI. The exclusion criteria were: (I) abstracts, case reports, editorials, or review articles; (II) studies involving animal models; (III) studies that did not report the number of patients at risk in their KM curves; or (IV) studies without sufficient data on the impact of CI on exercise performance. When multiple published reports from the same study were available, the report with the most comprehensive outcome details was selected.

### 2.3 Data Extraction

Four independent researchers (M.B., P.D., M.M., and R.N‐J.) conducted the data extraction process from the selected studies. The following information was extracted from each eligible study: publication year, study design, study location, total sample size, populations' age and sex distribution, inclusion of patients with atrial fibrillation, percentage of each HF phenotype in the population, proportion of patients using β‐blockers, type of exercise testing modality, method for evaluating CI, CI cut‐off point, and baseline left ventricular ejection fraction. Any discrepancies in the extracted data were resolved after discussions with a third investigator (K.T.).

### 2.4 Definitions and Outcomes

CI was mostly defined as the inability to achieve a predetermined cutoff point using two established formulas. The first was failure to reach the age‐predicted maximal heart rate, calculated as 220 minus age, during peak exercise [2]. The second formula was based on heart rate (HR) reserve, defined as the ratio of the difference between peak exercise HR and resting HR to the difference between the age‐predicted maximal HR and resting HR [13].

The primary outcome was overall prognosis, defined as the duration from study enrollment to occurrence of composite cardiovascular events (myocardial infarction, HF rehospitalization, left ventricular assist device implantation, heart transplantation, all‐cause mortality), besides death from any cause alone. Secondary outcomes included resting HR, peak HR during exercise, HR reserve (defined as the difference between resting and peak HR), exercise duration, peak exercise capacity, peak oxygen consumption (VO_2_ max), and ventilatory efficiency slope (VE/VCO_2_ slope).

### 2.5 Quality Assessment

Two authors (P.D. and M.K.) independently evaluated the risk of bias utilizing the Cochrane Risk of Bias in Non‐randomized Studies‐of Exposure (ROBINS‐E) [14]. Any disagreements were settled through mutual consensus. Additionally, the quality of evidence for each outcome measure was evaluated using the Grading of Recommendations, Assessment, Development, and Evaluations (GRADE) framework [15].

### 2.6 Statistical Analysis

Continuous variables were expressed as means ± standard deviation or medians (interquartile ranges), while categorical data were shown as numbers and percentages. To assess overall prognosis among HF patients, we reconstructed individual patient data (IPD) from KM curves for our primary outcomes following the methodology proposed by Liu et al [16]. In brief, the KM curves were digitized by importing survival (*Y*‐axis) and time (*X*‐axis) values from the original KM curves utilizing the “Shiny application.” The extracted values were merged with the number of patients at risk in CI and non‐CI groups during the specified time points on the KM curve to recreate the IPD. The accuracy of every reconstructed IPD was assessed using root mean square error, mean absolute error, and maximum absolute error statistics. All reconstructed IPD sets were merged into one database, and the aggregated survival curves and Cox proportional hazards model were fitted to estimate Hazard ratios and 95% confidence intervals (CIs).

The Grambsch–Therneau test and Schoenfeld residual plots were used to assess the proportional hazards assumption for the Cox model. Additionally, to quantify the loss of life expectancy, restricted mean survival time (RMST) was calculated [17]. Differences in RMST between the CI and non‐CI groups were compared over the time where at least 20% of participants remained (2500 days) [18].

To compare exercise performance between patients with HF based on the presence or absence of CI, we used mean differences (MDs) with 95% confidence intervals (CIs) by applying the DerSimonian and Laird random‐effects model. Heterogeneity was assessed using Higgins and Thompson's *I* ^2^ statistic and between‐study variance (τ^2^), with the Sidik–Jonkman estimator employed to calculate τ^2^.

Sensitivity analyses were conducted using a leave‐one‐out approach. Moreover, potential sources of heterogeneity were further explored through pre‐specified subgroup analyses based on HF phenotype (HFrEF vs. HFpEF), exercise testing modality (treadmill vs. cycle ergometry), age (inclusion vs. exclusion of participants aged < 60 years), and inclusion of participants with atrial fibrillation. The subgroup analysis by exercise testing modality was performed given previous evidence suggesting that cycle ergometry activates less muscle mass and yields lower heart rate and peak VO_2_ responses [19].

Meta‐regression analyses were performed using a mixed‐effects model to assess the potential influence of the proportion of the male participants and β‐blocker use on exercise performance outcomes, with visualized results using bubble plots. Publication bias was evaluated through inspection of funnel plot asymmetry and Egger's test for outcomes with more than 10 studies. Moreover, the “trim and fill” method was used to adjust the funnel plot asymmetry and recalculate the results in the presence of publication bias [20]. All statistical analyses were conducted using the *IPDfromKM*, *dmetar*, *meta*, *survival*, *survminer*, and *ggplot2* packages in R version 4.1.3 (The R Foundation, Vienna, Austria) [21]. All *p*‐values were two‐sided, and values < 0.05 were considered significant.

## 3 Results

### 3.1 Baseline Characteristics

Database searches identified 4948 citations, of which 3363 unique records remained after duplicate removal. Following title and abstract screening, 3260 records were excluded. Full texts of 103 articles were reviewed, resulting in the inclusion of 22 articles representing 21 unique studies in the final analysis (Figure 1).

**Figure 1:**
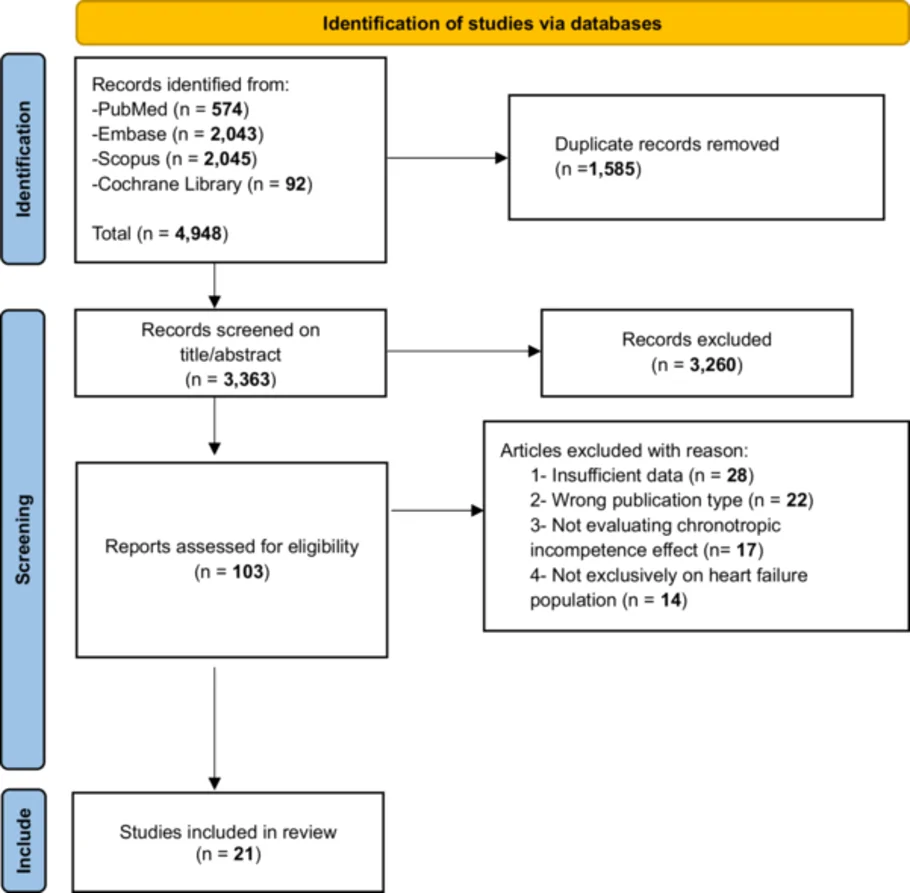
PRISMA flow chart.

Among the included studies, 11 were retrospective cohorts [6, 9, 10, 13, 22, 23, 24, 25, 26, 27, 28], eight were prospective cohorts [11, 12, 29, 30, 31, 32, 33, 34, 35], and two were randomized controlled trials [36, 37]. Among the 5604 patients with HF, 2022 had CI. Detailed characteristics of the included studies are provided in Table 1.

**Table 1:** Characteristics of the included studies.

Study, year	Study type	Country of origin	Total sample size	Inclusion of < 60 participants	Male sex (%)	Inclusion of AF	HFrEF (%)	Beta blocker use (%)	Ejection fraction	Method used for evaluating CI	CI cut‐off point	Exercise testing method	CI (n)	Outcomes
Clark, 1995 [13]	Post‐hoc study	UK	57	Yes	N/A	Yes	100.0	0	29.6 ± 15.2	HRR	0.8	Treadmill	16	Exercise time, peak VO_2_
Fei, 1996 [29]	Prospective cohort	UK	41	Yes	75.6	No	100.0	0	24 ± 10	HRR	0.8	Treadmill	10	Resting HR, maximum HR, HR reserve, peak VO_2_
Roche, 2001 [22]	Post‐hoc study	France	21	Yes	85.7	No	100.0	0	29 ± 8	HRR	0.8	Cycle ergometry	14	Resting HR, maximum HR, HR reserve, peak VO_2_
Brubaker, 2006 [6]	Post‐hoc study	USA	102	No	47	No	56.9	N/A	N/A	HRR	0.8	Cycle ergometry	23	Maximum HR, peak exercise output, peak VO_2_
Witte, 2006 [23]	Post‐hoc study	UK	237	Yes	75.1	No	100.0	69.2	32.4 ± 7.9	HRR	0.8	Treadmill	103	Survival outcomes, peak VO_2_, VE/VCO_2_ slope
Jorde, 2008 [30]	Prospective cohort	USA	278	Yes	76.7	No	100.0	72.4	N/A	HRR	0.8	Treadmill	128	Resting HR, maximum HR, HR reserve, peak VO_2_
Al‐Najjar, 2012 [24]	Post‐hoc study	UK	411	Yes	81.4	Yes	100.0	65.5	N/A	HRR	0.8	Treadmill	174	Survival outcomes, resting HR, maximum HR, HR reserve, exercise time, peak VO_2_, VE/VCO_2_ slope
Magri, 2012 [25]	Post‐hoc study	Italy	639	Yes	84	Yes	100.0	85.9	35.4 ± 8.2	HRR	0.8	Cycle ergometry	115	Resting HR, maximum HR, HR reserve, peak VO_2_
Benes, 2013 [31]	Prospective cohort	Czech Republic	81	Yes	85.2	No	100.0	97	24 ± 6	HRR	0.38	Cycle ergometry	20	Survival outcomes, resting HR, maximum HR, HR reserve, peak exercise output, peak VO_2_, VE/VCO_2_ slope
Magri, 2014 [26]	Post‐hoc study	Italy	1045	Yes	81	No	100.0	82	28 ± 7	HRR	0.65	Cycle ergometry	328	Survival outcomes, Resting HR, maximum HR, HR reserve, peak VO_2_, VE/VCO_2_ slope
Mohammed, 2014 [36]	Randomized clinical trial	USA	215	Yes	51.6	No	0	76.3	60 [55–66]	HRR	0.8	Treadmill and Cycle ergometry	51	Peak VO_2_
Hajdusek, 2016 [34]	Prospective cohort	Czech Republic	80	Yes	85.9	No	100.0	97.4	23.5 ± 6.3	MCR	0.66	Cycle ergometry	58	Survival outcomes, Resting HR, peak VO_2_, VE/VCO_2_ slope
Jamil, 2016 [9]	Post‐hoc study	UK	214	Yes	68.7	Yes	100.0	N/A	> 35	HRR	0.8	Treadmill	107	Exercise time, peak VO_2_, VE/VCO_2_ slope
Klein, 2016 [32]	Prospective cohort	USA	108	Yes	30	Yes	0	58	61 ± 7	HRR	0.8	Cycle ergometry	27	Resting HR, maximum HR, HR reserve, exercise time, peak exercise output, peak VO_2_, VE/VCO_2_ slope
Shen, 2017 [33]	Prospective cohort	China	26	Yes	76.9	No	0	96.2	43.9 ± 5.7	APMHR	0.7	Treadmill	13	Resting HR, maximum HR, HR reserve
Santos, 2020 [27]	Post‐hoc study	USA	718	Yes	66	Yes	61.0	83	34 ± 15	HRR	0.6	Cycle ergometry	180	Survival outcomes, resting HR, maximum HR, HR reserve, peak VO_2_, VE/VCO_2_ slope
Sarma, 2020 [11]	Prospective cohort	USA	13	No	46	No	0	92	> 50%	APMHR	0.8	Treadmill	7	Maximum HR, peak VO_2_
Karvonen, 2024 [37]	Randomized clinical trial	Finland, Germany	46	No	65.2	Yes	N/A	63	40.8 ± 11.8	APMHR	0.8	N/A	11	Resting HR
Magri, 2024 [28]	Post‐hoc study	Italy	864	Yes	79	No	100.0	85	43.3 ± 2.8	HRR	0.5	Cycle ergometry	430	Survival outcomes
Tashiro, 2024 [12]	Prospective cohort	Japan	129	Yes	72.9	No	0	75.2	51 [46‐6]	HRR	0.8	Cycle ergometry	68	Survival outcomes, resting HR, maximum HR, HR reserve, peak exercise output, peak VO_2_, VE/VCO_2_ slope
Yuasa, 2024 [10]	Post‐hoc study	Japan	246	Yes	43	Yes	0	31.9	62.9 ± 7.6	HRR	0.8	Cycle ergometry	112	Survival outcomes, resting HR, maximum HR, HR reserve, exercise time, peak exercise output, peak VO_2_, VE/VCO_2_ slope
Lin, 2025 [35]	Prospective cohort	Taiwan	113	Yes	46.9	Yes	0	59.3	> 50%	MCR	0.8	Cycle ergometry	85	Survival outcomes, resting HR, maximum HR, HR reserve, peak VO_2_, VE/VCO_2_ slope

Abbreviations: AF, atrial fibrillation; APMHR, age‐predicted maximal heart rate; CI, chronotropic incompetence; HFrEF, heart failure with reduced ejection fraction; HR, heart rate; HRR, heart rate reserve; MCR, metabolic chronotropic relation.

### 3.2 Prognosis Outcomes

A total of six studies, comprising 1331 patients (704 with CI and 627 without CI), were included in the IPD‐reconstructed meta‐analysis for overall prognosis [10, 12, 27, 31, 34, 35]. Over a follow‐up duration of 8 years (3000 days) with a median follow‐up of 3 years (1130 days), CI was associated with a significantly higher risk of composite cardiovascular events (Hazard ratio: 2.75; 95% CI: 2.18 to 3.48; *p* < 0.001; Figure 2A). The proportional hazards assumption was met (Grambsch–Therneau test *p*: 0.23; Supporting Information S1: Figure 1A). RMST analysis also revealed that over 2500 days, patients with CI experienced 410 fewer event‐free days compared to their non‐CI counterparts (1757 days, 95% CI: 1682 to 1833 vs. 2167 days, 95% CI: 2105 to 2229: *p* < 0.001; Figure 2B).

**Figure 2:**
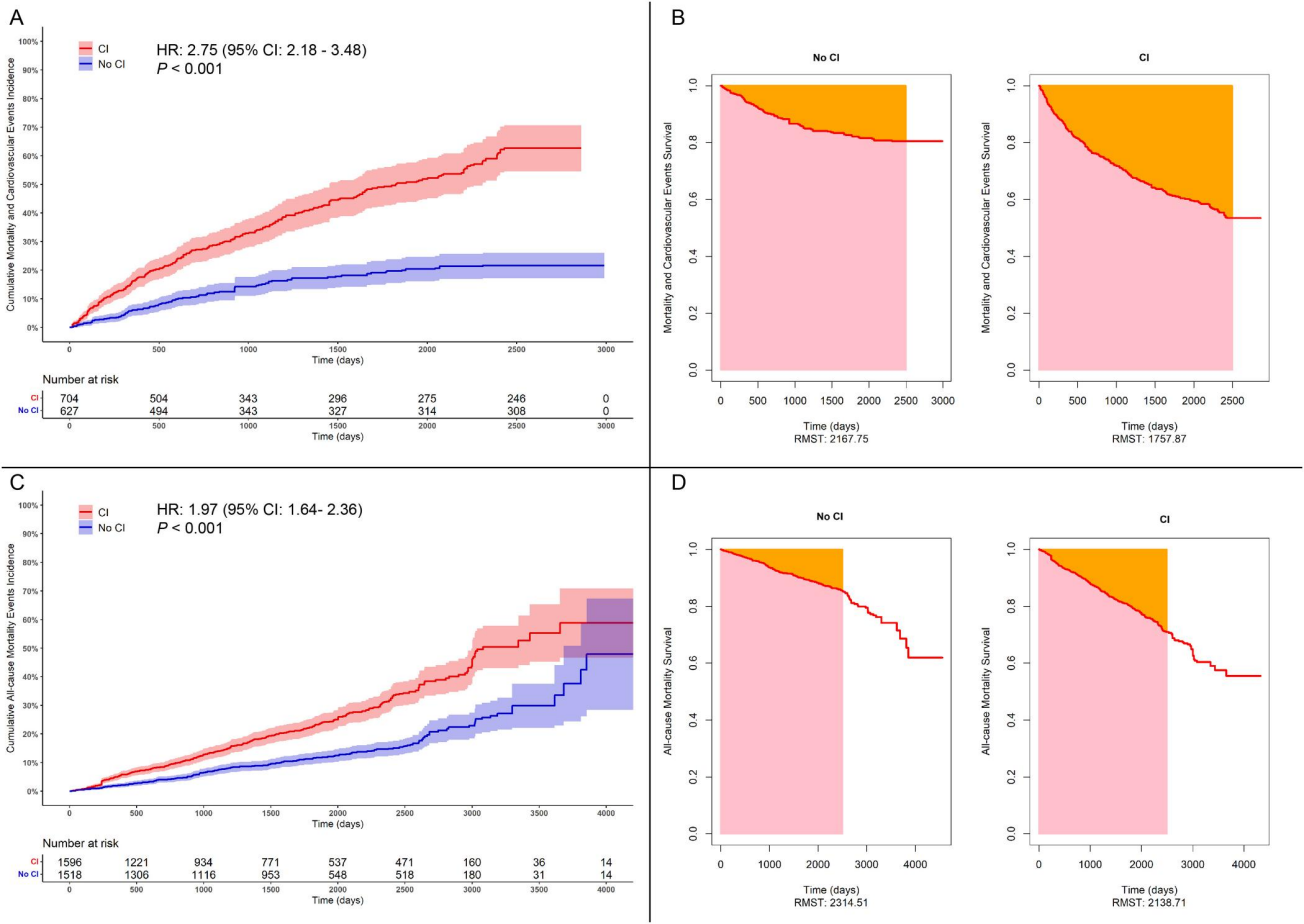
(A) Reconstructed Kaplan–Meier curve and (B) Restricted mean survival time (RMST) of overall prognosis in CI and non‐CI patients. (C) Reconstructed Kaplan–Meier curve and (D) RMST of all‐cause mortality in CI and non‐CI patients.

For all‐cause mortality, five studies comprising 3114 patients (1596 with CI and 1518 without CI) were analyzed [23, 24, 26, 27, 28]. Over 12 years (4400 days) of follow‐up with a median of 4.5 years (1708 days), the survival rate was 41.1% in the CI group and 62% in the non‐CI group. CI was associated with a 97% increased risk of all‐cause mortality (Hazard ratio: 1.97; 95% CI: 1.64 to 2.36; *p* < 0.001; Figure 2C). The proportional hazards assumption was met (Grambsch–Therneau test *p*: 0.22; Supporting Information S1: Figure 1B). RMST analysis indicated 176 fewer event‐free days in the CI group over 2500 days (2314 days, 95% CI: 2285 to 2343 vs. 2138 days, 95% CI: 2099 to 2177: *p* < 0.001; Figure 2D).

### 3.3 Exercise Performance

The pooled analysis demonstrated that patients with CI had a lower resting HR (MD: −6.25 bpm, 95% CI: −8.83 to −3.66, *I* ^2^ = 82.1%; *p* < 0.001; Figure 3A). Similarly, patients with CI had a lower peak HR during exercise (MD: −35.12 bpm, 95% CI: −38.11 to −32.13, *I* ^2^ = 88.7%; *p* < 0.001; Figure 3B). The pooled analysis also showed that patients with CI had a blunted HR reserve compared to the non‐CI subgroup (MD: −28.84 bpm, 95% CI: −31.66 to −26.02, *I* ^2^ = 91.6%; *p* < 0.001; Figure 3C).

**Figure 3:**
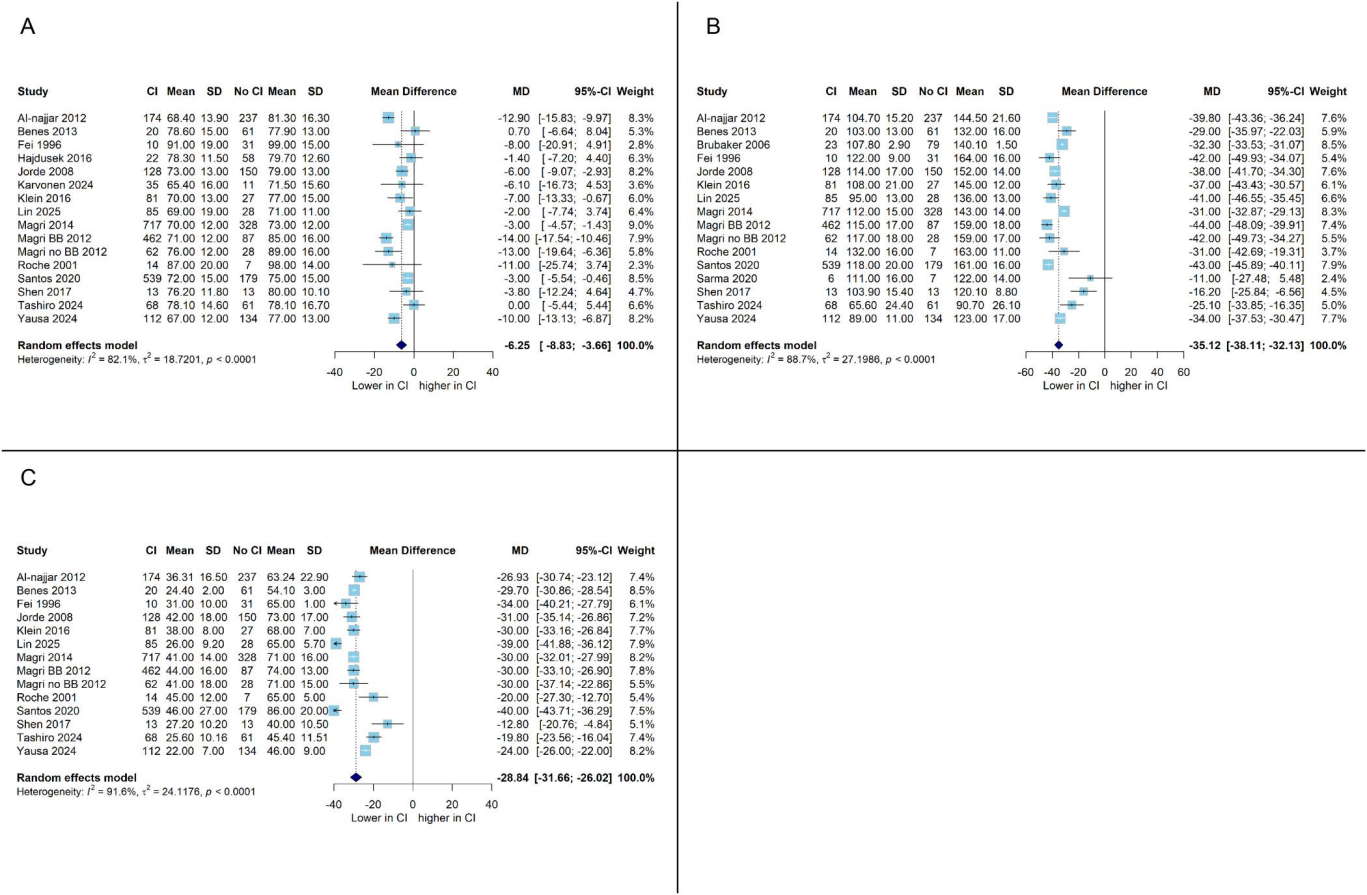
Forest plots comparing (A) resting HR, (B) peak HR, and (C) HR reserve between CI and non‐CI patients. CI, chronotropic incompetence; HR, heart rate; MD, mean difference; SD, standard deviation.

The pooled analysis demonstrated that patients with CI had significantly shorter exercise durations (MD: −77.68 s, 95% CI: −143.65 to −11.72, *I* ^2^ = 92.2%; *p*: 0.021; Figure 4A). Moreover, patients with CI had significantly lower peak workout performance than those without CI (MD: −19.15 watts, 95% CI: −23.46 to −14.85; *I* ^2^ = 51.3%; *p* < 0.001; Figure 4B).

**Figure 4:**
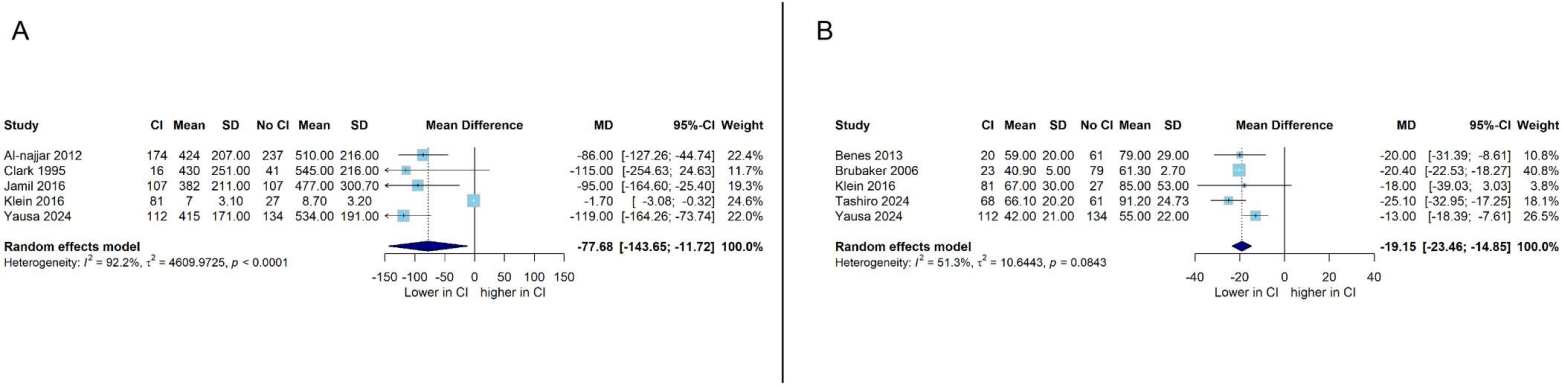
Forest plots comparing (A) exercise duration, and (B) peak exercise capacity between CI and non‐CI patients. CI, chronotropic incompetence; HR, heart rate; MD, mean difference; SD, standard deviation.

The pooled analysis demonstrated that patients with CI had significantly lower peak VO_2_ during exercise (MD: −3.34 mL kg^−1^ min^−1^, 95% CI: −3.98 to −2.69, *I* ^2^ = 83.2%; *p* < 0.001; Figure 5A). Additionally, VE/VCO_2_ slope was significantly higher among patients with CI (MD: 2.20, 95% CI: 1.35 to 3.05, *I* ^2^ = 59.5%; *p* < 0.001; Figure 5B).

**Figure 5:**
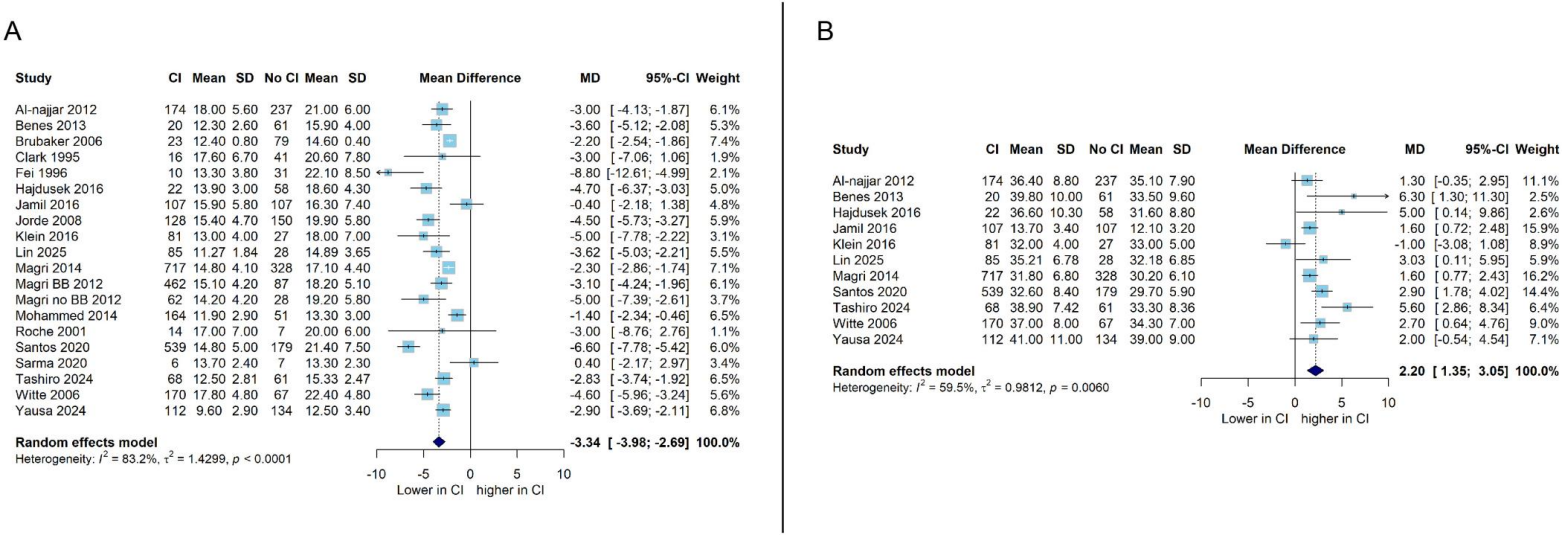
Forest plots comparing (A) peak VO_2_, and (B) VE/VCO_2_ slope between CI and non‐CI patients. CI, chronotropic incompetence; HR, heart rate; MD, mean difference; SD, standard deviation.

### 3.4 Sensitivity Analyses

Accuracy assessments of IPD confirmed high precision in data extraction across all included studies, as provided in Supporting Information S2: Table 3. Sensitivity analyses also indicated that exclusion of any single study did not affect the results for prognostic outcomes (Supporting Information S1: Figure 2). Likewise, the effect of CI on resting HR, peak HR, and HR reserve remained robust after sequential exclusion of individual studies (Supporting Information S1: Figure 3). However, omitting the study conducted by Klein et al. eliminated between‐study heterogeneity for exercise duration (MD: −100.59, 95% CI: −127.98 to −73.20, *I* ^2^ = 0%) [32], indicating that this study accounted for most of the observed heterogeneity (Supporting Information S1: Figure 4). Leave‐one‐out analyses further supported the robustness of findings for both peak VO_2_ and VE/VCO_2_ slope, with consistent effect estimates across iterations (Supporting Information S1: Figure 5).

### 3.5 Subgroup Analyses

Among the studies reporting composite cardiovascular events, two studies, comprising 161 patients (139 with CI and 22 without CI), only enrolled patients with HFrEF [31, 34], while three studies comprising 452 patients (336 with CI and 116 without CI) only included HFpEF patients [10, 12, 35], and one study included both HFrEF and HFpEF patients [27]. Over a follow‐up duration of up to 8 years (3000 days), HFrEF patients with CI experienced a 91% increased risk of composite cardiovascular events (Hazard ratio: 1.91; 95% CI: 1.26 to 2.88; *p* < 0.001). Similarly, in the HFpEF population, CI was associated with a 177% increased risk of composite cardiovascular events over a follow‐up period of approximately 7 years (2500 days) (Hazard ratio: 2.77; 95% CI: 1.61 to 4.79; *p* < 0.001). For all‐cause mortality, all studies except one enrolled HFrEF patients [27], precluding subgroup analysis.

No significant differences in exercise performance were observed between patients with HFrEF and HFpEF as provided in Supporting Information S2: Table 4. Additionally, the mode of exercise testing (treadmill vs. cycle ergometry), age group, or the presence of atrial fibrillation did not significantly affect performance outcomes as summarized in Supporting Information S2: Tables 5–7.

### 3.6 Meta‐Regression

Meta‐regression analyses were performed to assess whether the proportion of male participants or β‐blocker use modified exercise performance outcomes. Nevertheless, no significant interaction was observed between the prevalence of male participants or β‐blocker use and exercise performance measures (Supporting Information S1: Figures 6–10).

### 3.7 Publication Bias

The inspection of funnel plots and Egger's test revealed no evidence of publication bias for resting HR (Egger's test *p*: 0.58), peak HR (Egger's test *p*: 0.51), HR reserve (Egger's test *p*: 0.79), and VE/VCO_2_ slope (Egger's test *p*: 0.19) (Supporting Information S1: Figures 11 and 12). The inspection of the funnel plot for peak VO_2_ revealed potential publication bias (Egger's test *p*: 0.04). Nevertheless, the “trim and fill” test indicated that the observed results remained significant after the data from the missing studies were filled (MD: −2.58 mL kg^−1^ min^−1^, 95% CI: −3.21 to −1.81, *I* ^2^ = 87.7%; *p* < 0.001; Supporting Information S12: Figure 13).

### 3.8 Quality Assessment

Risk of bias assessment categorized 19 studies (90.4%) as having a low risk of bias, while two studies (9.6%) have a moderate risk (Supporting Information S1: Figures 14 and 15). The overall quality of evidence for the study outcomes was rated as moderate, primarily due to the high heterogeneity between included studies, their observational design, and the potential for residual confounding, as provided in Supporting Information S2: Table 8.

## 4 Discussion

By combining the data from 21 studies, the present study reports the following key findings: (I) HF patients with CI exhibited poorer prognosis compared to those without CI, with approximately 1‐year shorter event‐free survival over a 7‐year follow‐up. This survival disadvantage was consistent across both HFrEF and HFpEF subgroups. (II) CI was associated with a twofold higher risk of all‐cause mortality and nearly half a year fewer event‐free days during 7‐year‐follow‐up. (III) CI was correlated with poorer exercise capacity, reflected in shorter exercise durations and reduced peak exercise output. (IV) CI patients also exhibited poorer functional efficiency during exercise, characterized by lower peak VO_2_, with a higher level of breathing discomfort, reflected in a higher VE/VCO_2_ slope. (V) Additionally, CI was linked to significantly lower peak HR and reduced HR reserve during exercise. With a sample size of nearly 5604 HF patients, this meta‐analysis represents the most comprehensive evaluation of the long‐term impact of CI on both prognosis and exercise performance in the HF population (Graphical Abstract).

In our analysis, HF patients with CI exhibited a 175% higher risk of adverse cardiovascular outcomes and a 97% higher risk of mortality. While CI has previously been identified as a predictor of adverse outcomes both in individuals without cardiovascular disease and patients with coronary artery disease [38, 39], its prognostic significance in HF populations is still not well recognized. Similar to our findings, a meta‐analysis of six studies reported 24% higher odds of mortality in HFrEF patients with CI (log Odds ratio: −1.24, 95% CI: −2.20 to −0.28; *p* = 0.01) [8]. However, their analysis relied on odds ratios, which do not account for time‐to‐event data. Given the importance of follow‐up durations in evaluating survival analysis, our analysis provided a more accurate estimate, as the hazard ratio incorporates the effect of time as well [40]. Our results also extend this evidence by demonstrating that CI is associated with poorer prognosis in both HFrEF and HFpEF subgroups.

Several mechanisms may explain the observed outcomes. HF patients with CI often experience a higher burden of metabolic risk factors [10], which are directly associated with poorer prognosis [41]. Moreover, HF patients with CI typically present with more advanced disease, indicated by worse New York Heart Association functional class compared to their non‐CI counterparts [10, 42]. The abnormal HR response to exercise in CI patients has also been directly associated with increased mortality risk [43]. Data from the Framingham Study demonstrated that failure to reach target heart rate during exercise was a strong predictor of mortality, independent of ST segment changes and other cardiovascular risk factors [6]. Other studies suggested that an abnormal heart rate response during exercise may be a stronger predictor of mortality than ischemic ST‐segment depression on electrocardiography evaluations [44]. Nevertheless, the causal role of CI as an independent predictor of poorer prognosis and mortality in HF remains uncertain, as some studies have not found CI to be independently associated with reduced survival after adjusting for other confounders [45].

We also found that CI was associated with poorer exercise capacity (demonstrated in exercise durations and reduced peak power output) and poorer functional efficiency (presented with peak VO_2_) during exercise. Several studies have demonstrated poorer exercise performance in HFrEF patients [46], as increases in cardiac output in HFrEF patients rely more on HR acceleration than on stroke volume or arteriovenous oxygen difference; therefore, CI is closely tied to exercise capacity in these patients [47]. Nevertheless, there are conflicting results regarding the effect of CI on functional efficiency in HFpEF patients. A meta‐analysis of 17 studies found that CI decreased peak VO_2_ in HFrEF patients (MD: −3.86 mL kg^−1^ min^−1^, 95% CI: −4.83 to −2.89, *p* < 0.01), however no difference was observed in HFpEF subgroup (MD: −0.73 mL kg^−1^ min^−1^, 95% CI: −3.78 to 2.32, *p*: 0.64) [8]. In contrast, we found that CI decreased peak VO_2_ in both HFrEF and HFpEF patients. Previous studies reported limited adaptations of stroke volume to compensatory mechanisms for increasing cardiac output during activity in the HFpEF population [11], as increases in HR may compromise left ventricular filling in these patients with prolonged relaxation. Therefore, CI may provide a protective mechanism for exercise efficiency in patients with HFpEF. However, HFpEF patients with CI have more severe symptoms with worse New York Heart Association functional class [10], which limits their exercise capacity as reflected by lower exercise intensity and shorter exercise time, leading to reduced peak VO_2_.

In our meta‐analysis, CI was also associated with poorer breathing efficiency during exercise (presented in higher VE/VCO_2_ slope). The observed increase in VE/VCO_2_ slope can be a consequence of pulmonary perfusion/ventilation mismatch attributed to the inability to augment cardiac output during exercise or increased pulmonary vascular tone due to sympathetic and parasympathetic nervous system imbalance that may adversely impact right ventricular function [48]. VE/VCO_2_ slope has been proven to be a potential predictor for adverse outcomes in HF patients [49].

Our study also demonstrated that age group, the presence of atrial fibrillation, exercise training modality (treadmill vs. cycle ergometry) and use of β‐blockers do not have an influence on exercise performance in the HF population. These findings are comparable to the findings of other studies demonstrating that use of β‐blockers does not affect peak VO_2_, exercise duration, or HR reserve [25, 47], supporting the idea that the effect of impaired chronotropic response on exercise outcomes of heart failure is an intrinsic component of the disease irrespective of β‐blockers use. Recent guidelines recommend β‐blockers prescription for HFrEF patients; however, its prescription in HFpEF patients is still debated [50].

## 5 Limitations

There are several limitations to this meta‐analysis. First, there is no standard definition of CI in the HF population due to the already mismatched cardiac output and metabolic demand. However, most studies have used the HR reserve formula to diagnose CI, which will account for different age groups despite simpler formulas [2]. Moreover, different cut‐off values were used in the included articles for diagnosing CI, which may play a role in the heterogeneity observed in the meta‐analyses. Second, most of the included studies were observational, which may have introduced selection bias and unaccounted confounding variables like physical activity status, potentially impacting the observed outcomes. It would be nice if these findings were supported in larger evaluations accounting for potential confounding variables. Third, long‐term follow‐up results need to be interpreted with extra caution, as the number of studies comparing survival outcomes between the two groups at follow‐ups of more than 4 years was limited. Nevertheless, the results of this meta‐analysis are broadly applicable, as the study participants were derived from real‐world data across diverse countries.

## 6 Conclusions

In this meta‐analysis of 21 studies, CI was associated with poorer survival outcomes, as well as reduced physical capacity and poorer functional and ventilatory efficiency in both HFrEF and HFpEF populations. Our findings highlight the importance of diagnosis of CI in patients with HFrEF as well as HFpEF, and of conducting more evaluations of potential treatment strategies to reduce the harmful effects of CI and improve long‐term outcomes in this vulnerable population.

## Author Contributions

M.T., K.T., P.D., R.N.‐J, M.B., and M.M. were involved in the study design, drafting of the manuscript, and had a significant role in the development of the paper. P.D, R.N.‐J, A.H.B., and M.R. contributed to the development of the selection criteria, data extraction, and drafting of the manuscript. K.T. and M.M. developed the search strategy and performed the qualitative data synthesis and meta‐analysis. Z.H., M.K., A.F., and M.T. critically reviewed the manuscript. M.T. supervised the entire process. All authors participated in the critical review, provided feedback, and approved the final manuscript.

## Funding

The authors have nothing to report.

## Ethics Statement

The analyses relied exclusively on summary data from previously published studies, therefore, additional ethical approval was not required.

## Conflicts of Interest

The authors declare no conflict of interest.

## Disclosure of AI Programs

We utilized AI tools, specifically ChatGPT 4o and Grammarly GO, to aid in rephrasing portions of this article. The purpose was to improve clarity and readability only. Nevertheless, every piece of AI‐generated content was carefully reviewed and verified by the authors to ensure accuracy and integrity.

## Supporting information


Supporting File 1

Supporting File 2

## Data Availability

The data that support the findings of this study are available from the corresponding author upon reasonable request.
