# Supplementary material for: Chronotropic Incompetence Predicts Reduced Survival and Exercise Capacity in Heart Failure: A Meta‐Analysis of Reconstructed Time‐to‐Event Data
Source: Clin Cardiol. 2026 Nov 9;49(11):e70522. doi: 10.1002/clc.70522 (PMC13748947; doi:10.1002/clc.70522)
Supplement: Supplementary file 1 [file CLC-49-e70522-s001.docx]

# Supplementary Tables

**Supplementary Table 1.** Checklist of compliance with PRISMA guidelines.	2

**Supplementary Table 2.** Search strategy.	6

**Supplementary Table 3**. Accuracy assessment of reconstructed IPDs.	9

**Supplementary Table 4.** Subgroup analysis for exercise performance outcomes based on heart failure phenotype.	11

**Supplementary Table 5.** Subgroup analysis for exercise performance outcomes based on exercise testing modality.	13

**Supplementary Table 6.** Subgroup analysis for exercise performance outcomes based on age.	15

**Supplementary Table 7.** Subgroup analysis for exercise performance outcomes based on atrial fibrillation.	16

**Supplementary Table 8.** Quality of evidence according to GRADE framework.	17

| **Section and Topic** | **Item #** | **Checklist item** | **Location where item is reported** |
| --- | --- | --- | --- |
| **TITLE** | | |  |
| Title | 1 | Identify the report as a systematic review. | Reported on title page. |
| **ABSTRACT** | | |  |
| Abstract | 2 | See the PRISMA 2020 for Abstracts checklist. | Abstract provided by PRISMA 2020 abstracts checklist and the Journal’s instructions to authors. |
| **INTRODUCTION** | | |  |
| Rationale | 3 | Describe the rationale for the review in the context of existing knowledge. | Rationale for conducting this study is provided in the second paragraph of ‘Introduction’. |
| Objectives | 4 | Provide an explicit statement of the objective(s) or question(s) the review addresses. | Explicitly stated in the second paragraph of ‘Introduction’. |
| **METHODS** | | |  |
| Eligibility criteria | 5 | Specify the inclusion and exclusion criteria for the review and how studies were grouped for the syntheses. | Explained under ‘Eligibility Criteria’, respectively. |
| Information sources | 6 | Specify all databases, registers, websites, organisations, reference lists and other sources searched or consulted to identify studies. Specify the date when each source was last searched or consulted. | Reported under ‘Search Strategy and Screening’. |
| Search strategy | 7 | Present the full search strategies for all databases, registers and websites, including any filters and limits used. | Provided in Supplementary Table 2. |
| Selection process | 8 | Specify the methods used to decide whether a study met the inclusion criteria of the review, including how many reviewers screened each record and each report retrieved, whether they worked independently, and if applicable, details of automation tools used in the process. | Explained under ‘Search Strategy and Screening’. |
| Data collection process | 9 | Specify the methods used to collect data from reports, including how many reviewers collected data from each report, whether they worked independently, any processes for obtaining or confirming data from study investigators, and if applicable, details of automation tools used in the process. | Reported under ‘Data Extraction’. |
| Data items | 10a | List and define all outcomes for which data were sought. Specify whether all results that were compatible with each outcome domain in each study were sought (e.g. for all measures, time points, analyses), and if not, the methods used to decide which results to collect. | Reported under ‘Data Extraction’. |
|  | 10b | List and define all other variables for which data were sought (e.g. participant and intervention characteristics, funding sources). Describe any assumptions made about any missing or unclear information. | Reported under ‘Data Extraction’. |
| Study risk of bias assessment | 11 | Specify the methods used to assess risk of bias in the included studies, including details of the tool(s) used, how many reviewers assessed each study and whether they worked independently, and if applicable, details of automation tools used in the process. | Reported under ‘Quality Assessment’. |
| Effect measures | 12 | Specify for each outcome the effect measure(s) (e.g. risk ratio, mean difference) used in the synthesis or presentation of results. | Reported under ‘Outcomes’. |
| Synthesis methods | 13a | Describe the processes used to decide which studies were eligible for each synthesis (e.g. tabulating the study intervention characteristics and comparing against the planned groups for each synthesis (item #5)). | Explained under ‘Search Strategy and Screening’. |
|  | 13b | Describe any methods required to prepare the data for presentation or synthesis, such as handling of missing summary statistics, or data conversions. | Reported under ‘Statistical Analysis’. |
|  | 13c | Describe any methods used to tabulate or visually display results of individual studies and syntheses. | Reported under ‘Statistical Analysis’. |
|  | 13d | Describe any methods used to synthesize results and provide a rationale for the choice(s). If meta-analysis was performed, describe the model(s), method(s) to identify the presence and extent of statistical heterogeneity, and software package(s) used. | Explained under ‘Statistical Analysis’. |
|  | 13e | Describe any methods used to explore possible causes of heterogeneity among study results (e.g. subgroup analysis, meta-regression). | Explained under ‘Statistical Analysis’. |
|  | 13f | Describe any sensitivity analyses conducted to assess robustness of the synthesized results. | Explained under ‘Statistical Analysis’. |
| Reporting bias assessment | 14 | Describe any methods used to assess risk of bias due to missing results in a synthesis (arising from reporting biases). | Reported under ‘Statistical Analysis’. |
| Certainty assessment | 15 | Describe any methods used to assess certainty (or confidence) in the body of evidence for an outcome. | Reported under ‘Quality Assessment’. |
| **RESULTS** | | |  |
| Study selection | 16a | Describe the results of the search and selection process, from the number of records identified in the search to the number of studies included in the review, ideally using a flow diagram. | Explained under ‘Study Characteristics’ and the flow diagram depicted in Figure 1. |
|  | 16b | Cite studies that might appear to meet the inclusion criteria, but which were excluded, and explain why they were excluded. | Explained under ‘Study Characteristics’ and the flow diagram depicted in Figure 1. |
| Study characteristics | 17 | Cite each included study and present its characteristics. | Reported under ‘Study Characteristics’. |
| Risk of bias in studies | 18 | Present assessments of risk of bias for each included study. | Provided in Supplementary Figures 9 and 10. |
| Results of individual studies | 19 | For all outcomes, present, for each study: (a) summary statistics for each group (where appropriate) and (b) an effect estimate and its precision (e.g. confidence/credible interval), ideally using structured tables or plots. | Provided in Figures 2-5. |
| Results of syntheses | 20a | For each synthesis, briefly summarise the characteristics and risk of bias among contributing studies. | Reported in Table 1 and Supplementary Figure 9 and 10. |
|  | 20b | Present results of all statistical syntheses conducted. If meta-analysis was done, present for each the summary estimate and its precision (e.g. confidence/credible interval) and measures of statistical heterogeneity. If comparing groups, describe the direction of the effect. | Reported under ‘Primary outcomes’, and ‘Secondary outcomes’ and depicted in Fig 2-5. |
|  | 20c | Present results of all investigations of possible causes of heterogeneity among study results. | Reported under ‘Survival Outcomes’, and ‘Exercise Performance’ and provided in Supplementary Figures. |
|  | 20d | Present results of all sensitivity analyses conducted to assess the robustness of the synthesized results. | Reported under ‘Survival Outcomes’, and ‘Exercise Performance’ and provided in Supplementary Figures. |
| Reporting biases | 21 | Present assessments of risk of bias due to missing results (arising from reporting biases) for each synthesis assessed. | Reported under ‘Survival Outcomes’, and ‘Exercise Performance’ and provided in Supplementary Figures. |
| Certainty of evidence | 22 | Present assessments of certainty (or confidence) in the body of evidence for each outcome assessed. | Reported under ‘Quality assessment’. |
| **DISCUSSION** | | |  |
| Discussion | 23a | Provide a general interpretation of the results in the context of other evidence. | Provided in ‘Discussion’. |
|  | 23b | Discuss any limitations of the evidence included in the review. | Provided in ‘Discussion’. |
|  | 23c | Discuss any limitations of the review processes used. | Provided in ‘Limitations’. |
|  | 23d | Discuss implications of the results for practice, policy, and future research. | Provided in ‘Discussion’. |
| **OTHER INFORMATION** | | |  |
| Registration and protocol | 24a | Provide registration information for the review, including register name and registration number, or state that the review was not registered. | Provided in first paragraph of ‘Methods’. |
|  | 24b | Indicate where the review protocol can be accessed, or state that a protocol was not prepared. | Provided in first paragraph of ‘Methods’. |
|  | 24c | Describe and explain any amendments to information provided at registration or in the protocol. | No amendments were made. |
| Support | 25 | Describe sources of financial or non-financial support for the review, and the role of the funders or sponsors in the review. | Provided in title page. |
| Competing interests | 26 | Declare any competing interests of review authors. | Provided in cover letter. |
| Availability of data, code and other materials | 27 | Report which of the following are publicly available and where they can be found: template data collection forms; data extracted from included studies; data used for all analyses; analytic code; any other materials used in the review. | Data were provided from the included studies and are available upon reasonable request from the corresponding author. |

**Supplementary Table 1.** Checklist of compliance with PRISMA guidelines.

| **Dataset** | **Search Syntax** | **Result** |
| --- | --- | --- |
| PubMed | (("Heart failure"[Mesh] OR "Heart Failure, Diastolic"[Mesh] OR "Heart Failure, Systolic"[Mesh] OR "Cardiac Failure" OR "Heart Decompensation" OR "Decompensation, Heart" OR "Congestive Heart Failure" OR "Heart Failure, Congestive" OR "Heart Failure, Right-Sided" OR "Heart Failure, Right Sided" OR "Right-Sided Heart Failure" OR "Right Sided Heart Failure" OR "Heart Failure, Left-Sided" OR "Heart Failure, Left Sided" OR "Left-Sided Heart Failure" OR "Left Sided Heart Failure" OR "Myocardial Failure" OR "Diastolic Heart Failures" OR "Diastolic Heart Failure" OR "Heart Failure, Preserved Ejection Fraction" OR "Heart Failure, Normal Ejection Fraction" OR "HFpEF" OR "Heart Failures, Systolic" OR "Systolic Heart Failures" OR "Heart Failure, Reduced Ejection Fraction" OR "Systolic Heart Failure" OR "HFrEF"))  AND  (("Chronotropic incompetence" OR "Chronotropic response" OR "Impaired heart rate response" OR "Sick Sinus Syndrome" OR "Syndrome, Sick Sinus" OR "Sick Sinus Node Syndrome" OR "Sinus Node Dysfunction" OR "Dysfunction, Sinus Node" OR "Dysfunctions, Sinus Node" OR "Sinus Node Disease" OR "Sinus Node Diseases")) | 584 |
| Embase | ('heart failure'/exp OR 'diastolic heart failure'/exp OR 'systolic heart failure'/exp OR 'cardiac failure':ti,ab,kw OR 'heart decompensation':ti,ab,kw OR 'decompensation, heart':ti,ab,kw OR 'congestive heart failure':ti,ab,kw OR 'heart failure, congestive':ti,ab,kw OR 'heart failure, right-sided':ti,ab,kw OR 'heart failure, left-sided':ti,ab,kw OR 'myocardial failure':ti,ab,kw OR 'diastolic heart failures':ti,ab,kw OR 'heart failure, preserved ejection fraction':ti,ab,kw OR 'hfpef':ti,ab,kw OR 'heart failures, systolic':ti,ab,kw OR 'heart failure, reduced ejection fraction':ti,ab,kw OR 'hfref':ti,ab,kw)  AND  ('chronotropic incompetence':ti,ab,kw OR 'chronotropic response':ti,ab,kw OR 'impaired heart rate response':ti,ab,kw OR 'sick sinus syndrome':ti,ab,kw OR 'syndrome, sick sinus':ti,ab,kw OR 'sick sinus node syndrome':ti,ab,kw OR 'sinus node dysfunction':ti,ab,kw OR 'dysfunction, sinus node':ti,ab,kw OR 'dysfunctions, sinus node':ti,ab,kw OR 'sinus node disease':ti,ab,kw OR 'sinus node diseases':ti,ab,kw) | 2147 |
| Scopus | (TITLE-ABS-KEY("Heart failure" OR "Heart Failure, Diastolic" OR "Heart Failure, Systolic" OR "Cardiac Failure" OR "Heart Decompensation" OR "Decompensation, Heart" OR "Congestive Heart Failure" OR "Heart Failure, Congestive" OR "Heart Failure, Right-Sided" OR "Heart Failure, Left-Sided" OR "Myocardial Failure" OR "Diastolic Heart Failures" OR "Heart Failure, Preserved Ejection Fraction" OR "HFpEF" OR "Heart Failures, Systolic" OR "Heart Failure, Reduced Ejection Fraction" OR "HFrEF"))  AND  (TITLE-ABS-KEY("Chronotropic incompetence" OR "Chronotropic response" OR "Impaired heart rate response" OR "Sick Sinus Syndrome" OR "Syndrome, Sick Sinus" OR "Sick Sinus Node Syndrome" OR "Sinus Node Dysfunction" OR "Dysfunction, Sinus Node" OR "Dysfunctions, Sinus Node" OR "Sinus Node Disease" OR "Sinus Node Diseases")) | 2120 |
| Cochrane Library | (MeSH descriptor: [Heart Failure] explode all trees) OR (MeSH descriptor: [Heart Failure, Systolic] explode all trees) OR (MeSH descriptor: [Heart Failure, Diastolic] explode all trees) OR ("Cardiac Failure" OR "Heart Decompensation" OR "Decompensation, Heart" OR "Congestive Heart Failure" OR "Heart Failure, Congestive" OR "Heart Failure, Right-Sided" OR "Heart Failure, Left-Sided" OR "Myocardial Failure" OR "Diastolic Heart Failures" OR "Heart Failure, Preserved Ejection Fraction" OR "HFpEF" OR "Heart Failures, Systolic" OR "Heart Failure, Reduced Ejection Fraction" OR "Systolic Heart Failure" OR "HFrEF")  AND  ("Chronotropic incompetence" OR "Chronotropic response" OR "Impaired heart rate response" OR "Sick Sinus Syndrome" OR "Syndrome, Sick Sinus" OR "Sick Sinus Node Syndrome" OR "Sinus Node Dysfunction" OR "Dysfunction, Sinus Node" OR "Dysfunctions, Sinus Node" OR "Sinus Node Disease" OR "Sinus Node Diseases") | 97 |
| Total |  | 4948 |

**Supplementary Table 2.** Search strategy.

| **Study** | **Subgroup** | **RMSE** | **Mean absolute error** | **Maximum absolute error** |
| --- | --- | --- | --- | --- |
| *Composite cardiovascular events* | | | | |
| Benes 2013 | No CI | 0.014 | 0.012 | 0.025 |
|  | CI | 0.006 | 0.004 | 0.008 |
| Hajdusek 2016 | No CI | 0.005 | 0.003 | 0.008 |
|  | CI | 0.013 | 0.010 | 0.022 |
| Lin 2025 | No CI | 0.003 | 0.009 | 0.011 |
|  | CI | 0.006 | 0.015 | 0.003 |
| Santos 2020 | No CI | 0.001 | 0.001 | 0.002 |
|  | CI | 0.001 | 0.001 | 0.002 |
| Tashiro 2024 | No CI preserved O2 | 0.006 | 0.006 | 0.002 |
|  | CI preserved O2 | 0.003 | 0.010 | 0.017 |
|  | No CI impaired O2 | 0.006 | 0.003 | 0.016 |
|  | CI impaired O2 | 0.017 | 0.016 | 0.018 |
| Yausa 2024 | No CI | 0.007 | 0.005 | 0.007 |
|  | CI | 0.005 | 0.004 | 0.009 |
| *All-cause mortality* | | | | |
| Al-Najjar 2022 | No CI without BB | 0.003 | 0.002 | 0.005 |
|  | CI without BB | 0.006 | 0.005 | 0.01 |
|  | No CI with BB | 0.002 | 0.002 | 0.004 |
|  | CI with BB | 0.002 | 0.002 | 0.004 |
| Magri 2014 | No CI | 0.005 | 0.004 | 0.014 |
|  | CI | 0.003 | 0.002 | 0.005 |
| Magri 2024 | No CI | 0.001 | <0.001 | 0.002 |
|  | CI | 0.001 | <0.001 | 0.003 |
| Santos 2020 | No CI | 0.001 | 0.001 | 0.002 |
|  | CI | 0.001 | 0.001 | 0.002 |
| Witte 2006 | No CI | 0.006 | 0.005 | 0.010 |
|  | CI | 0.007 | 0.005 | 0.015 |

**Supplementary Table 3**. Accuracy assessment of reconstructed IPDs. BB, β-blockers; CI, Chronotropic incompetence; RMSE, root mean square error.

| **Outcome** | **Subgroup** | **Number of studies** | **MD** | **95% CI** | **I^2^** | **P value of interaction** |
| --- | --- | --- | --- | --- | --- | --- |
| Resting HR | HFrEF | 10 | -7.53 | -11.55 to -3.51 | 87.8% | 0.17 |
|  | HFpEF | 5 | -4.87 | -9.29 to -0.46 | 69.4% |  |
|  | Both | 1 | -3 | -5.53 to -0.46 | -- |  |
| Peak HR | HFrEF | 8 | -37.30 | -41.79 to -32.80 | 87.3% | 0.35 |
|  | HFpEF | 6 | -29.38 | -36.47 to -22.89 | 84.0% |  |
|  | Both | 2 | -37.56 | -48.05 to -27.08 | 97.8% |  |
| HR reserve | HFrEF | 8 | -29.51 | -30.95 to -28.06 | 37.2% | 0.74 |
|  | HFpEF | 5 | -25.57 | -32.99 to -18.14 | 96.2% |  |
|  | Both | 1 | -40.00 | -43.71 to -36.29 | -- |  |
| Exercise time | HFrEF | 2 | -88.32 | -127.89 to -48.76 | 0.0% | 0.69 |
|  | HFpEF | 3 | -68.92 | -158.80 to 20.95 | 96.0% |  |
| Peak exercise capacity | HFrEF | 1 | -20 | -31.39 to -8.61 | -- | 0.92 |
|  | HFpEF | 3 | -18.49 | -27.95 to -9.04 | 67.8% |  |
|  | Both | 1 | -20.4 | -22.53 to -18.27 | -- |  |
| Peak VO_2_ | HFrEF | 11 | -3.85 | -4.68 to -3.02 | 68.9% | 0.19 |
|  | HFpEF | 7 | -2.28 | -3.24 to -1.32 | 72.8% |  |
|  | Both | 2 | -4.36 | -8.67 to -0.05 | 98.0% |  |
| VE/VCO_2_ slope | HFrEF | 5 | 2.11 | 1.01 to 3.20 | 34.2% | 0.55 |
|  | HFpEF | 5 | 2.05 | 0.26 to 3.85 | 73.6% |  |
|  | Both | 1 | 2.90 | 1.78 to 4.01 | -- |  |

**Supplementary Table 4.** Subgroup analysis for exercise performance outcomes based on heart failure phenotype. CI, Confidence interval; HFpEF, Heart failure with preserved ejection fraction; HFrEF, Heart failure with reduced ejection fraction; MD, Mean difference.

| **Outcome** | **Subgroup** | **Number of studies** | **MD** | **95% CI** | **I^2^** | **P value of interaction** |
| --- | --- | --- | --- | --- | --- | --- |
| Resting HR | Treadmill | 5 | -8.24 | -13.29 to -3.18 | 74.8% | 0.38 |
|  | Cycle ergometry | 11 | -5.61 | -8.63 to -2.58 | 82.8% |  |
| Peak HR | Treadmill | 5 | -31.96 | -39.78 to -24.15 | 87.3% | 0.37 |
|  | Cycle ergometry | 11 | -35.81 | -39.17 to -32.45 | 89.8% |  |
| HR reserve | Treadmill | 4 | -26.74 | -33.41 to -20.06 | 84.9% | 0.46 |
|  | Cycle ergometry | 10 | -29.50 | -32.79 to 26.21 | 93.3% |  |
| Exercise time | Treadmill | 3 | -89.95 | -124.35 to -55.56 | 0.0% | 0.60 |
|  | Cycle ergometry | 2 | -58.07 | -172.94 to 56.78 | 96.1% |  |
| Peak exercise capacity | Cycle ergometry | 5 | -19.15 | -23.45 to -14.85 | -- | -- |
| Peak VO_2_ | Treadmill | 7 | -3.23 | -4.86 to -1.59 | 82.1% | 0.70 |
|  | Cycle ergometry | 13 | -3.58 | -4.42 to -2.83 | 86.1% |  |
| VE/VCO_2_ slope | Treadmill | 3 | 1.68 | 0.95 to 2.41 | 0.0% | 0.24 |
|  | Cycle ergometry | 8 | 2.57 | 1.25 to 3.90 | 74.3% |  |

**Supplementary Table 5.** Subgroup analysis for exercise performance outcomes based on exercise testing modality. CI, Confidence interval; MD, Mean difference.

| **Outcome** | **Subgroup** | **Number of studies** | **MD** | **95% CI** | **I^2^** | **P value of interaction** |
| --- | --- | --- | --- | --- | --- | --- |
| Resting HR | Participants <60 years included | 15 | -6.25 | -8.91 to -3.59 | 83.3% | 0.98 |
|  | Participants <60 years excluded | 1 | -6.1 | -16.7 to 4.53 | -- |  |
| Peak HR | Participants <60 years included | 14 | -35.91 | -39.41 to -32.42 | 87.7% | 0.24 |
|  | Participants <60 years excluded | 2 | -23.30 | -43.92 to -2.68 | 84.3% |  |
| HR reserve | Participants <60 years included | 14 | -28.84 | -31.66 to -26.02 | 91.6% | -- |
| Exercise time | Participants <60 years included | 5 | -77.68 | -143.65 to -11.72 | 92.2% | -- |
| Peak exercise capacity | Participants <60 years included | 4 | -18.68.15 | -25.66 to -11.69 | 53.6% | 0.64 |
|  | Participants <60 years included | 1 | -20.40 | -22.53 to -18.27 | -- |  |
| Peak VO_2_ | Participants <60 years included | 18 | -3.59 | -4.32 to -2.86 | 81.3% | 0.71 |
|  | Participants <60 years excluded | 2 | -1.22 | -3.69 to 1.24 | 74.2% |  |
| VE/VCO_2_ slope | Participants <60 years included | 11 | 2.20 | 1.35 to 3.05 | 59.5% | -- |

**Supplementary Table 6.** Subgroup analysis for exercise performance outcomes based on age. CI, Confidence interval; MD, Mean difference.

| **Outcome** | **Subgroup** | **Number of studies** | **MD** | **95% CI** | **I^2^** | **P value of interaction** |
| --- | --- | --- | --- | --- | --- | --- |
| Resting HR | AF participants included | 8 | -8.70 | -12.43 to -4.97 | 84.1% | **0.01*** |
|  | AF participants excluded | 8 | -3.32 | -4.87 to -1.77 | 8.6% |  |
| Peak HR | AF participants included | 7 | -40.10 | -43.14 to -37.07 | 70.1% | **<0.01*** |
|  | AF participants excluded | 9 | -31.03 | -34.21 to -27.85 | 78.5% |  |
| HR reserve | AF participants included | 7 | -31.42 | -36.47 to -26.37 | 94.1% | 0.10 |
|  | AF participants excluded | 7 | -26.22 | -29.81 to 22.62 | 88.1% |  |
| Exercise time | AF participants included | 5 | -77.68 | -143.65 to -11.72 | 92.2% | -- |
| Peak exercise capacity | AF participants included | 2 | -13.31 | -18.52 to -8.91 | 0.0% | **0.01*** |
|  | AF participants excluded | 3 | -20.70 | -22.72 to -18.68 | 0.0% |  |
| Peak VO_2_ | AF participants included | 9 | -3.60 | -4.76 to -2.45 | 82.0% | 0.46 |
|  | AF participants excluded | 11 | -3.09 | -3.84 to -2.34 | 81.0% |  |
| VE/VCO_2_ slope | AF participants included | 6 | 1.66 | 0.63 to 2.69 | 58.2% | 0.09 |
|  | AF participants excluded | 5 | 3.56 | 1.63 to 5.49 | 66.7% |  |

**Supplementary Table 7.** Subgroup analysis for exercise performance outcomes based on atrial fibrillation. AF, atrial fibrillation; CI, Confidence interval; MD, Mean difference.

| **Outcome** | **No. of studies** | **Study design** | **Quality of evidence** | | | | | **Summary of findings** | | | **Quality** |
| --- | --- | --- | --- | --- | --- | --- | --- | --- | --- | --- | --- |
|  |  |  | Risk of bias | Inconsistency | Indirectness | Imprecision | Publication bias | HR/MD (95% CI) | P-value | Plausible  confounding |  |
| *Prognosis Outcomes* | | | | | | | | | | | |
| Composite cardiovascular events | 6 | Mostly observational | Not serious | Not serious | Not serious | Not serious | Not serious | 2.75; (2.18 to 3.48) | < 0.001 | Plausible | Moderate |
| All-cause mortality | 5 | Mostly observational | Not serious | Serious ^a^ | Not serious | Not serious | Not serious | 1.97; (1.64 to 2.36) | < 0.001 | Plausible | Moderate |
| *Exercise Performance* | | | | | | | | | | | |
| Resting HR | 16 | Mostly observational | Not serious | Serious ^a^ | Not serious | Not serious | Not serious | -6.25 (-8.83 to -3.66) | < 0.001 | Plausible | Moderate |
| Peak HR | 16 | Mostly observational | Not serious | Serious ^a^ | Not serious | Not serious | Not serious | -35.12 (-38.11 to -32.13) | < 0.001 | Plausible | Moderate |
| HR reserve | 14 | Mostly observational | Not serious | Serious ^a^ | Not serious | Not serious | Not serious | -28.84 (-31.66 to -26.02) | < 0.001 | Plausible | Moderate |
| Exercise time | 5 | Mostly observational | Not serious | Not serious | Not serious | Serious ^b^ | Not serious | -77.68 (-143.65 to -11.72) | 0.021 | Plausible | Moderate |
| Peak exercise capacity | 5 | Mostly observational | Not serious | Not serious | Not serious | Serious ^b^ | Not serious | -19.15 (-23.46 to -14.85) | < 0.001 | Plausible | Moderate |
| Peak VO₂ | 20 | Mostly observational | Not serious | Serious ^a^ | Not serious | Not serious | Not serious | -3.34 (-3.98 to -2.69) | < 0.001 | Plausible | Moderate |
| VE/VCO₂ slope | 11 | Mostly observational | Not serious | Serious ^a^ | Not serious | Not serious | Not serious | 2.20 (1.35 to 3.05) | < 0.001 | Plausible | Moderate |

**Supplementary Table 8.** Quality of evidence according to GRADE framework.

a. I^2^ scores are high for the variable, suggesting significant heterogeneity between the included studies. Moreover, our subgroup analyses were unable to find the source of the heterogeneity.
b. Limited number of studies and infrequent incidence of outcomes made the results less precise.
