# Supplementary material for: Chronotropic Incompetence Predicts Reduced Survival and Exercise Capacity in Heart Failure: A Meta‐Analysis of Reconstructed Time‐to‐Event Data
Source: Clin Cardiol. 2026 Nov 9;49(11):e70522. doi: 10.1002/clc.70522 (PMC13748947; doi:10.1002/clc.70522)
Supplement: Supplementary file 2 [file CLC-49-e70522-s002.docx]

# Supplementary Figures

**Supplementary Figure 1.** Schoenfeld residual plots for evaluating Cox hazard models for (A) overall prognosis (B) all-cause mortality.	3

**Supplementary Figure 2.** Leave-one-out analysis comparing (A) overall prognosis (B) all-cause mortality between CI and non-CI patients. CI, Chronotropic incompetence.	4

**Supplementary Figure 3.** Leave-one-out analysis comparing (A) resting heart rate, (B) peak heart rate, and (C) heart rate reserve between CI and non-CI patients. CI, Chronotropic incompetence.	5

**Supplementary Figure 4.** Leave-one-out analysis comparing (A) exercise duration, and (B) peak exercise capacity between CI and non-CI patients.	6

**Supplementary Figure 5.** Leave-one-out analysis comparing (A) peak VO₂, and (B) VE/VCO₂ slope between CI and non-CI patients.	7

**Supplementary Figure 6.** Bubble plot of the modifying effect of β-blocker use on (A) resting heart rate, (B) peak heart rate, and (C) heart rate reserve.	8

**Supplementary Figure 7.** Bubble plot of the modifying effect of β-blocker use on (A) peak VO₂, and (B) VE/VCO₂ slope.	9

**Supplementary Figure 8.** Bubble plot of the modifying effect of the proportion of male participants on (A) resting heart rate, (B) peak heart rate, and (C) heart rate reserve.	10

**Supplementary Figure 9.** Bubble plot of the modifying effect of the proportion of male participants on (A) exercise duration, and (B) peak exercise capacity.	11

**Supplementary Figure 10.** Bubble plot of the modifying effect of the proportion of male participants on (A) peak VO₂, and (B) VE/VCO₂ slope.	12

**Supplementary Figure 11.** Funnel plot of the association between CI and (A) resting heart rate, (B) peak heart rate, and (C) heart rate reserve.	13

**Supplementary Figure 12.** Funnel plot of the association between CI and (A) peak VO₂, and (B) VE/VCO₂ slop.	14

**Supplementary Figure 13.** Trim and fill analysis of the association between CI and peak VO₂. CI, Chronotropic incompetence.	15

**Supplementary Figure 14.** Traffic light plot evaluating risk of bias of the included studies.16

**Supplementary Figure 15.** Summary plot evaluating risk of bias of the included studies. 17

**Supplementary Figure 1.** Schoenfeld residual plots for evaluating Cox hazard models for (A) overall prognosis (B) all-cause mortality.

**Supplementary Figure 2.** Leave-one-out analysis comparing (A) overall prognosis (B) all-cause mortality between CI and non-CI patients. CI, Chronotropic incompetence.

**Supplementary Figure 3.** Leave-one-out analysis comparing (A) resting heart rate, (B) peak heart rate, and (C) heart rate reserve between CI and non-CI patients. CI, Chronotropic incompetence.

**Supplementary Figure 4.** Leave-one-out analysis comparing (A) exercise duration, and (B) peak exercise capacity between CI and non-CI patients. CI, Chronotropic incompetence.

**Supplementary Figure 5.** Leave-one-out analysis comparing (A) peak VO₂, and (B) VE/VCO₂ slope between CI and non-CI patients. CI, Chronotropic incompetence.

**Supplementary Figure 6.** Bubble plot of the modifying effect of β-blocker use on (A) resting heart rate, (B) peak heart rate, and (C) heart rate reserve.

**Supplementary Figure 7.** Bubble plot of the modifying effect of β-blocker use on (A) peak VO₂, and (B) VE/VCO₂ slope.

**Supplementary Figure 8.** Bubble plot of the modifying effect of the proportion of male participants on (A) resting heart rate, (B) peak heart rate, and (C) heart rate reserve.

**Supplementary Figure 9.** Bubble plot of the modifying effect of the proportion of male participants on (A) exercise duration, and (B) peak exercise capacity.

**Supplementary Figure 10.** Bubble plot of the modifying effect of the proportion of male participants on (A) peak VO₂, and (B) VE/VCO₂ slope.

**Supplementary Figure 11**. Funnel plot of the association between CI and (A) resting heart rate, (B) peak heart rate, and (C) heart rate reserve. CI, Chronotropic incompetence.

**Supplementary Figure 12**. Funnel plot of the association between CI and (A) peak VO₂, and (B) VE/VCO₂ slope. CI, Chronotropic incompetence.

**Supplementary Figure 13**. Trim and fill analysis of the association between CI and peak VO₂. CI, Chronotropic incompetence.

**Supplementary Figure 14.** Traffic light plot evaluating risk of bias of the included studies.

**Supplementary Figure 15.** Summary plot evaluating risk of bias of the included studies.
